# MaReS (Magdeburg Reflective Writing Scoring Rubric for Feedback) – development of a feedback method for reflective writing in health professions education: A pilot study in veterinary medicine

**DOI:** 10.3205/zma001752

**Published:** 2025-04-15

**Authors:** Sabine Ramspott, Ulrike Sonntag, Anja Härtl, Stefan Rüttermann, Doris Roller, Marianne Giesler, Linn Hempel

**Affiliations:** 1 Trillium GmbH Medizinischer Fachverlag, Grafrath, Germany; 2 Charité – Universitätsmedizin Berlin, Institute of General Practic, Berlin, Germany; 3 University Hospital Augsburg, Department of Hygiene and Environmental Medicine, Augsburg, Germany; 4 Institut für medizinische und pharmazeutische Prüfungsfragen (IMPP), Mainz, Germany; 5 Ruprecht-Karls-University, Center for Psychosocial Medicine, Institute of Medical Psychology, Heidelberg, Germany; 6 Freiburg/Br., Germany; 7 Martin-Luther-Universität Halle-Wittenberg, Medical Faculty, Dorothea Erxleben Lernzentrum, Halle/Saale, Germany

**Keywords:** reflection, reflective writing, analytic scoring rubric, validity, feedback

## Abstract

**Aim:**

The aim of the study was to develop a scoring rubric that provides valuable feedback to students and to gather evidence for its construct validity.

**Methodology:**

The Magdeburg Reflective Writing Feedback and Scoring Rubric (MaReS) was developed in an iterative process following a symposium on reflection by a committee of the “DACH Association for Medical Education (GMA)” in June 2016.

25 essays written by 13 veterinary students were assessed by three independent raters with MaReS and by two raters with the REFLECT rubric in two runs (13 and twelve essays). Validity evidence was gathered referring to the following of Messick’s components of construct validity: content (rubric development), response process (rater manual, rater training, rating time, students’ evaluation), internal structure (inter-rater reliability, IRR), and relationship to other variables (comparison of the rating with the REFLECT rubric and a global rating scale).

**Results:**

The analytic rubric comprises twelve items that are rated on three-point rating scales. The authors developed an assignment with guiding questions for students and a rater manual. Results for free marginal kappa of the items of MaReS ranged from -0.08 to 0.77 for the first set of reflective essays and from 0.13 to 0.75 for the second set. Correlations between MaReS and the REFLECT rubric were positive (first run: r=0.92 (p<0.001); second run: r=0.29 (p=0.37)).

**Conclusion:**

MaReS might be a useful tool to guide students’ reflective writing and provide structured feedback in health professions education. Using more essays for a rater training and more training cycles are likely to result in higher IRRs.

## Zusammenfassung

**Zielsetzung:**

Das Ziel der Studie war die Entwicklung eines Bewertungsinstruments, mit dem Studierenden konstruktives Feedback gegeben werden kann sowie Belege für die Konstruktvalidität des Instruments zu sammeln.

**Methodik:**

Die Magdeburger Reflexionsskala (MaReS) wurde in einem iterativen Prozess im Anschluss an einen Workshop zum Thema Reflexion durch eine Arbeitsgruppe eines Ausschusses der „Gesellschaft für Medizinische Ausbildung (GMA)“ im Juni 2016 entwickelt.

25 Berichte von 13 Studierenden der Veterinärmedizin wurden in zwei Durchgängen (13 und zwölf Berichte) von drei unabhängigen Beurteilenden mit MaReS und von zwei Beurteilenden mit dem Instrument REFLECT bewertet. Die Validität wurde anhand der folgenden Komponenten der Konstruktvalidität von Messick ermittelt: Inhalt (Entwickeln des Instruments), Antwortprozess (Kodierleitfaden, Schulung der Beurteilenden, Bewertungsdauer, Evaluation durch Studierende), interne Struktur (Inter-Rater-Reliabilität, IRR) und Beziehung zu anderen Variablen (Vergleich der Bewertung mit Instrument REFLECT und einer globalen Bewertungsskala).

**Ergebnisse:**

Das analytische Instrument umfasst zwölf Items, die auf einer dreistufigen Skala bewertet werden. Die Autor:innen entwickelten eine Aufgabenstellung mit Leitfragen für die Studierenden sowie einen Kodierleitfaden. Die Ergebnisse für das freie marginale Kappa der Items von MaReS reichten von -0,08 bis 0,77 für die erste Gruppe von Reflexionsberichten und von 0,13 bis 0,75 für die zweite Gruppe. Die Korrelationen zwischen MaReS und REFLECT waren positiv (erster Durchlauf: r=0,92 (p<0,001); zweiter Durchlauf: r=0,29 (p=0,37)).

**Schlussfolgerung:**

MaReS kann ein nützliches Instrument sein, um das reflektierende Schreiben von Studierenden in der Ausbildung von Gesundheitsberufen sowohl anzuleiten als auch strukturiertes Feedback zu geben. Die Verwendung von weiteren Berichten zur Schulung der Beurteilenden und mehr Trainingszyklen würden vermutlich zu höheren IRR führen.

## 1. Introduction and aim

### 1.1. Reflection in health professions education

To improve patient care different roles that physicians [16] have to comply with and a set of domains for the veterinary practice [5] have been defined. In order to meet the requirements of these roles or domains healthcare professionals have to acquire many corresponding competences during their studies [https://www.avma.org/education/accreditation-policies-and-procedures-avma-council-education-coe], [13], [16], [36], [45]. As demands and knowledge grow, their working environment becomes more and more complex. To provide up-to-date healthcare, physicians and veterinarians consequently have to engage in life-long learning, where the ability to reflect on one’s own actions is an integral part [29]. Reflection was already regarded as an essential means of cognition in the Enlightenment (cf. 18^th^ century Kant, Fries, among others) and was described as “inner introspection”, among other things [17], [25]. Today’s literature describes reflection inter alia as an “in-depth consideration of events or situations: the people involved, what they experienced, and how they felt about it” [6]. 

Reflection therefore also supports the process of professional identity formation, a key development process for healthcare professions [9]. Furthermore, reflective practice seems to foster resilience [56] and to reduce stress [33]. These aspects gain more and more importance as the well-being of physicians and veterinarians has become an increasing concern in recent years [19], [31], [32], [55], [60].

The ability to reflect can be developed as part of a learning process [54] and fostered by different activities: Reflective writing has been used widely in medical education [27], [28], [46], [53], [58]. Other activity formats include group discussions [7], [33], online forums [35], digital storytelling [52] and collaborative drawing [34]. 

The effectiveness of “writing as a method” has been proven [48]. In writing down a perceived situation, people develop the awareness of what had happened and additionally train their expressive skills. This affects how one thinks, feels and acts. People who benefit the most are those who have little opportunity for self-disclosure in everyday life, who find it difficult to recognize and name feelings and who can try out perspectives for their fixed way of thinking while writing [54].

To promote reflection feedback can be a highly relevant and powerful tool, but a systematic review showed that in most of the studies using feedback as part of their intervention to develop reflection the methods and details of the feedback remained unclear [55]. Only one of the studies described the protocol that was used for providing feedback [3]. 

German speaking countries have many large medical and veterinary faculties with up to 870 first year students (e.g., LMU Munich, Germany). Reflective writing seems to be an ideal means to engage large numbers of students in reflection. Nevertheless, teaching reflection and providing feedback on reflective writings to a large number of students is expected to be a resource intensive endeavour. Individual written feedback is supposed to be more time consuming than the use of a rubric, where boxes can be ticked. Therefore, scoring rubrics can be a suitable means to simplify feedback. Since the use of structured reflection is relatively new in health professions education in Germany, Austria and Switzerland (DACH region), teachers are usually not very experienced in teaching this competency and providing feedback.

### 1.2. Scoring rubrics

Rubrics are often used to assess performance and reflective writing. There are two different ways of scoring: In holistic scoring the assessors rate the student’s essay as a complete unit against a prepared scale, whereas in analytic scoring the assessors break down the essay into its constituent elements, each of which is assigned a proportion of the available mark [22]. Educators usually use holistic scoring for large-scale assessment because it is assumed to be easy to handle, cheap and accurate. Analytical scoring is useful in teaching since the results can help teachers and students to identify students’ strengths and learning needs [24]. If the scoring of a reflective writing will be used as a formative assessment, the analytical approach seems more suitable.

Medical educators have developed several holistic [30], [44], [47], [51], [59], [61] and analytic [10], [11], [57] rubrics for the formative and/or summative assessment of reflective essays. Reis et al. developed “The Brown Educational Guide to the Analysis of Narrative” (BEGAN), a guide for crafting written feedback to students’ reflective writing [50]. This guide and the analytic rubrics can be used to provide feedback on students’ reflective essays. The REFLECT rubric comprises five major criteria (writing spectrum, presence, description of conflict or disorienting dilemma, attending to emotions, analysis and meaning making) and one optional minor criterion (attention to assignment). Four levels of reflection (habitual action, thoughtful action or introspection, reflection, and critical reflection) are assigned to these criteria. If the level critical reflection is achieved the learning outcomes are also to be defined as transformative or confirmatory learning [57]. The rubric of Devlin et al. comprises four dimensions (descriptive, comparative, personal and critical) with prompts (questions) to guide assessment and feedback [11]. Devi’s rubric is based on Koole’s indicators to describe the process of reflection [29] and Moon’s grading system [41]. Raters grade the essays from A to F [10]. So far, no assessment rubric for reflective writing in a medical context could be identified in German. 

### 1.3. Guides for reflective writing

There are several guides for reflective writing in medical education: In the appendix to his AMEE Guide, Sandars [51] gives examples for a student information sheet for undergraduate and postgraduate medical students based on Moon’s handbook [41], a template for structured reflection to develop a therapeutic relationship of professional practice after Johns [23] with 20 guiding questions and 11 questions to develop deeper reflection. Aronson et al. developed a reflective learning guide that consists of a structured approach to reflection based on the SOAP format with guiding questions and an information sheet about reflection and strategies for successful reflection. Aronson et al. tested their tool using a holistic scoring rubric [4]. We could not identify an analytic scoring rubric accompanied by guiding questions or a guide for students. 

### 1.4. Aim

The aim of the study was to develop a German analytic rubric for the formative assessment of reflective essays, that is aligned with guiding questions for students and provides valuable feedback to students. The use of the rubric should be easy and timesaving for teachers when assessing reflective essays. In addition, the authors’ goal was to gather validity evidence for the new tool. 

## 2. Methods

### 2.1. Scoring rubric development

The committee for Communicative and Social Competencies (KusK) of the “DACH Association for Medical Education (GMA)” (Gesellschaft für Medizinische Ausbildung, GMA) hosted a symposium on reflection in June 2016 to discuss possibilities to incorporate reflection into the teaching of communication and social skills for medical and veterinary students in Germany. The participants of one workshop examined three existing tools to assess reflective essays [44], [50], [57]. Based on these three tools, a first version of a scoring rubric was developed. 

After the workshop the scoring rubric was refined using Koole’s model of common elements describing the process of reflection [29] as a guide for the content selection. Concerning the structure and wording of the scoring rubric, Moskal’s recommendations for scoring rubric development were followed [43]. The feedback rubric was developed together with a matching assignment and guiding questions for students and a rater manual in an iterative process. After several revisions by the authors of this paper and after a first consensus was achieved, the rubric, assignment, and guiding questions, the rater manual and an example essay for reflective writing was sent to six external experts, who were engaged in reflective activities at their medical and veterinary faculties in Germany. The expert group comprised doctors, veterinarians, health scientists and psychologists. The feedback of the external experts on the tool and its elements was discussed and incorporated into the new version of the tool after consensus. The tool was named Magdeburg Reflective Writing Feedback and Scoring Rubric (Magdeburger Reflexionsskala, MaReS) according to its place of origin. 

### 2.2. Evidence for construct validity

**Figure 1:**
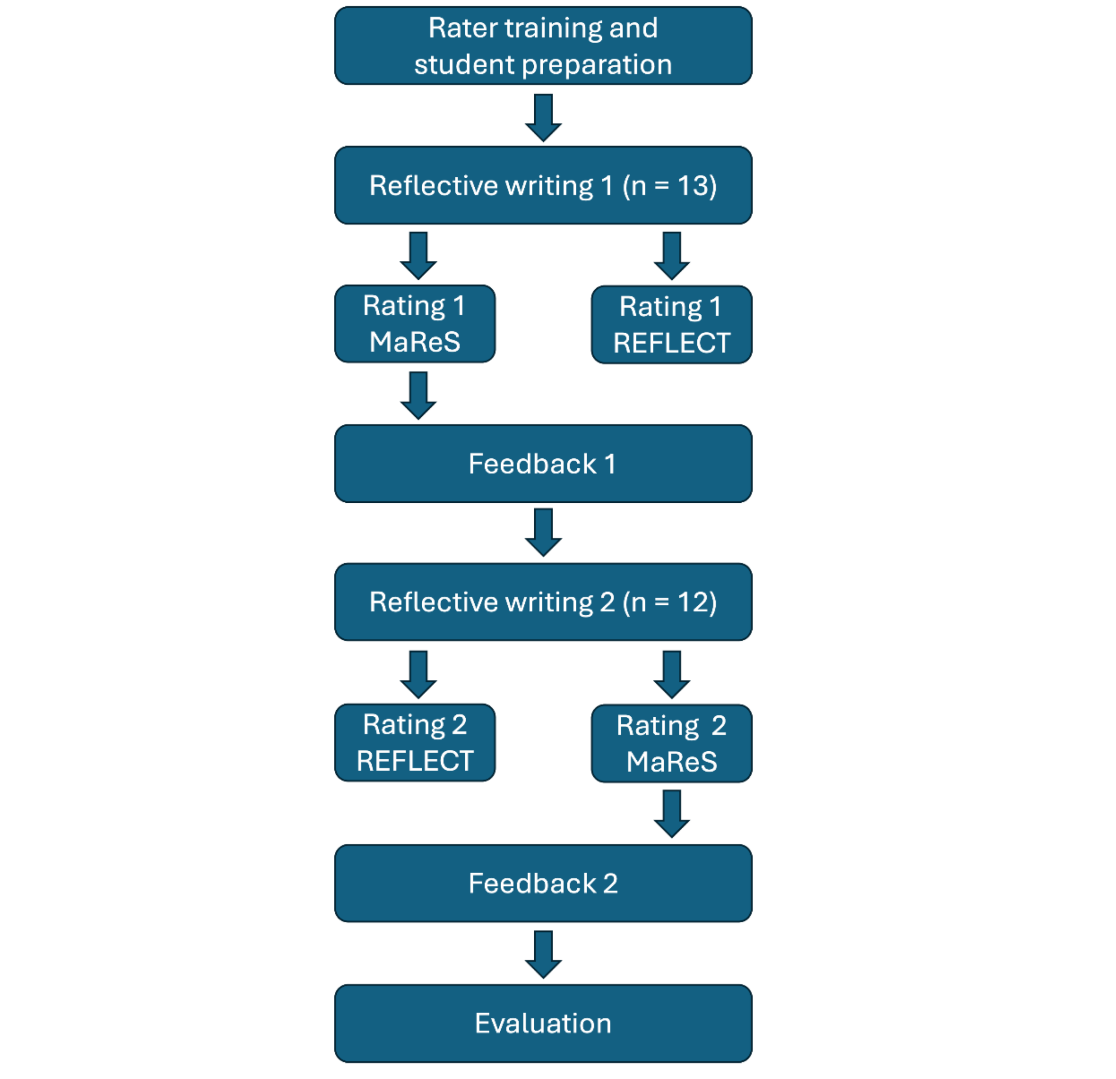
Study design MaReS

**Figure 2:**
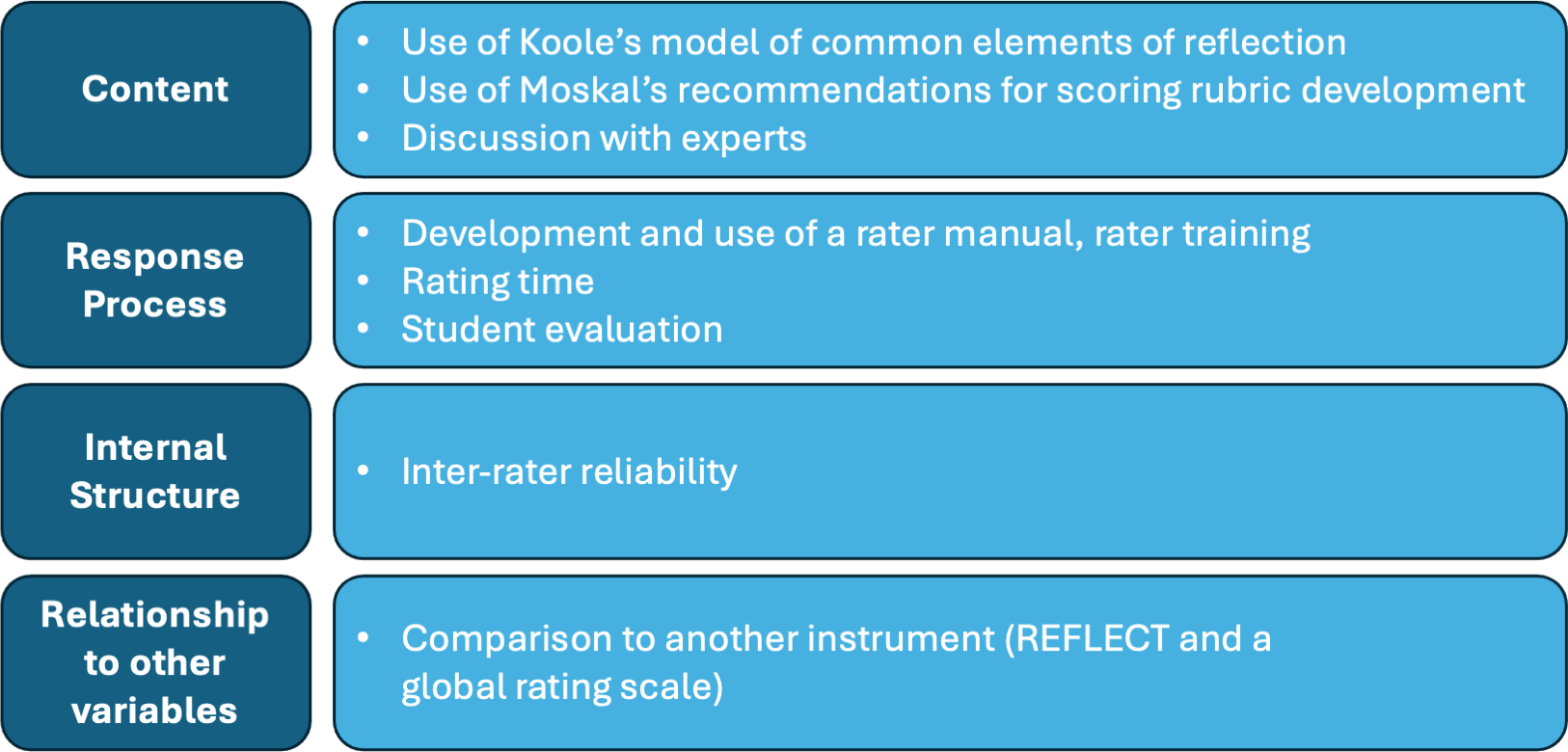
Sources of validity evidence for MaReS

“Validity is an integrated evaluative judgment of the degree to which empirical evidence and theoretical rationales support the adequacy and appropriateness of interpretations and actions based on test scores or other modes of assessment” [37]. The assessment tool itself cannot be declared valid (or not valid), but more (or less) validity evidence can be collected to support the proposed interpretations of assessment scores. The context in which the validity evidence is collected remains important: assessment data is more – or less – valid for a specific purpose, meaning or interpretation at a certain point of time and for a specific population for which validity evidence was collected [12]. In this study we collected validity evidence for using the rubric as a formative assessment tool of reflective capacity in reflective essays. The essays on veterinary practice were written by veterinary students who took part in an optional subject on reflection in 2017 and were rated by five raters who didn’t know the students; each essay was rated by three raters using the newly developed scoring rubric and two using the REFLECT rubric. We gathered validity evidence for four of the six sources of construct validity named by Messick [38]: content, response process, internal structure and relationship to other variables. Figure 1 (Fig. 1) shows the study design; figure 2 (Fig. 2) shows the sources of validity evidence selected for this study. In the following section we describe the methods that we used to obtain evidence for the different sources of construct validity in a chronological order. Therefore, the source “response process” is split up in this section.

#### 2.2.1. Content – feedback rubric development

The way in which we developed the instrument contributes to the evidence for content validity (see 2.1.). 

#### 2.2.2. Response process I – use of rater manual and rater training

All five raters attended the two-day KusK-Workshop on reflection in June 2016. After the development of MaReS, raters (us, lh, srü, dr, ah) were provided with the final version of the rubric, assignment, and guiding questions and the rater manual. They rated an example essay individually. Ratings were compared and divergent ratings were discussed with all raters in a telephone conference. Subsequently all raters agreed on a common approach for the items. The rater manual was adjusted accordingly. 

The same approach was followed for the REFLECT rubric [58] that we employed to evaluate the relationship to other variables (see 2.3.). Since there was no rater manual at that time, adjusting the manual did not apply. We used the English version of REFLECT and the four steps for its application [57]. 

#### 2.2.3. Internal structure – inter-rater reliability and relationship to other variables – comparison to another scoring rubric

The Ethics Committee of the LMU Munich granted ethical approval for the study (project number 17-065). Thirteen students of an elective course about reflection on veterinary practice each wrote one reflective essay after six hours of instruction about reflection in general and different reflective activities, including reflective writing based on MaReS. Students were made familiar with the assessment tool. They discussed different examples of reflective writing and the scoring with MaReS. The assignment was to *write a reflective essay about a concrete situation. The students had to choose a situation connected to their studies or occupation in which they have felt challenged during the interaction with a patient, a patient owner, a fellow student or a teacher.* The 13 essays were assessed by three raters using MaReS (ah, dr, lh) and by two raters using REFLECT (us, srü). One of the MaReS ratings was reported back to the students. Subsequently twelve of the students wrote another reflective essay that was rated with MaReS by three raters again (us, srü, lh) and with REFLECT by two raters (ah, dr). One of the MaReS ratings was again reported back to the students. All raters filled in a five-point scale overall rating for every essay. We used the overall and REFLECT rating for study purposes only and not reported back to the students. 

#### 2.2.4. Response process II – rating time and rater notes

Raters also reported the time they needed for their rating, and, if necessary, made notes on important aspects of the essay, reported difficult items and possible new anchor examples for the rater manual. 

#### 2.2.5. Response process III – student evaluation

After receiving two feedback sheets on their essays with MaReS, eight students filled in a questionnaire. It comprised five general questions on reflection and the elective subject, six questions on the assignment and guiding questions, four questions on the feedback they received and five questions on their reflective ability and views on reflective writing. 

### 2.3. Statistical methods

The rating time for the essays was analysed by descriptive statistics using mean values and standard deviation (SD). Inter-rater reliability (IRR) was determined using free marginal kappa [49]. Correlation between overall MaReS score (sum of the overall rating for MaReS of 3 raters), overall REFLECT score (sum of the overall rating for REFLECT of 2 raters) and global rating (sum of the corresponding global rating of 3 raters for MaReS and of 2 raters for REFLECT) was determined using Spearman’s Rank correlation coefficient. 

Students’ evaluations and raters’ feedback were analysed using descriptive statistics (absolute numbers) for questions with answers on a five-point scale and using content analysis according to Mayring for open questions. 

## 3. Results

### 3.1. Scoring rubric development

We developed a writing assignment with nine guiding questions and a rubric with twelve items that were assessed with a three-point scale (see attachment 1 ). The rater manual consisted of a short description of how to rate the essay and descriptors and anchors for all items and consecutive scales. 

### 3.2. Evidence for construct validity

#### 3.2.1. Content – MaReS development

See 3.1. 

#### 3.2.2. Response process – rating time

The number of characters (including spaces) of the reflective essays ranged from 2,765 to 8,488 with a mean of 5,359 characters (SD 1,660). The mean time that was needed to rate an essay with MaReS was 13.9 minutes with a standard deviation of 10.9. The mean rating time, i.e., time to read and rate the essay differed greatly between raters, ranging from a mean of 6.7 minutes (SD 2.5) to 28.5 minutes (SD 13.1). The mean duration of the REFLECT ratings was 7.8 minutes with a standard deviation of 5.2. Mean rating times for the individual raters ranged from 4.5 minutes (SD 1.2) to 14.5 (SD 6.9). 

#### 3.2.3. Internal structure – inter-rater reliability

Results for free marginal kappa of the items of MaReS ranged from -0.08 to 0.77 for the first set of reflective essays and from 0.13 to 0.75 for the second set (see table 1 (Tab. 1)). Results for free marginal kappa of the individual criterions of REFLECT ranged from -0.26 to 0.31 for the first set of reflective essays and from -0.11 to 0.38 for the second set (see table 2 (Tab. 2)). Free marginal kappa for the global rating of all five raters was 0.16 for the first set of essays and 0.22 for the second set of essays.

#### 3.2.4. Relationship to other variables – correlation between ratings

For essays 1 to 13 Spearman’s rank correlation coefficient for MaReS and the raters corresponding overall rating was r=0.43 (p=0.14), r=0.92 (p<0.001) for MaReS and REFLECT and r=0.83 (p<0.001) for REFLECT and global rating. 

For essays 14 to 25 Spearman’s Rank correlation coefficient for MaReS and the raters’ corresponding overall rating was r=0.75 (p=0.005), r=0.29 (p=0.37) for MaReS and REFLECT and r=0.87 (p<0.001) for REFLECT and the raters’ corresponding global rating.

#### 3.2.5. Response process – students’ evaluations

Eight students participated in the evaluation. The results of the closed questions are shown in table 2 (Tab. 2). All students rated their own writing competence positive and did understand the assignment. The difficulty of the assignment (“writing a reflection report on a self-selected challenging situation”) was rated differently by the students. For the first reflection report, most students rated their own selection of situations as “perfectly suitable” or “suitable”. For the second reflection report, the selection was rated as “partially suitable” by most students. 

All eight students rated the guiding questions as “very helpful” or “helpful”. Knowing that they will receive feedback on their reflection report was mostly rated “conducive” or “rather conducive”. When writing the second reflection report, half of the students felt “slightly more confident”, while the other half felt “slightly less confident”. Students rated their own ability to reflect mainly good (four students). Four students stated that they found the second reflection more difficult because they had difficulty finding a suitable situation to reflect on.

The structured feedback was rated positively by five students. They wrote that the feedback was non-judgmental, constructive, and helpful. At the same time one student criticized the fact that the feedback only related to the process of reflection and not to the situation. Thinking about their own ability to reflect, five students stated that they thought they are very self-critical and that this makes reflection difficult. Other responses where: not being able to think of a solution immediately; need for more opinions from others; more time to reflect. 

The content analysis revealed the following: In their free text responses, several students cited stress management/reduction as an expected personal benefit of reflection. Additionally personal development in various fields, improved understanding of and communication with others (including in private), benefits for future action in general and in learning situations were mentioned. 

Additionally, all free-text responses to three questions about the impact of the writing of and the feedback on reflection reports can be found in table 3 (Tab. 3). 

## 4. Discussion

### 4.1. Scoring rubric development

Practicality was our main priority for MaReS. The aim was to develop a scoring rubric that was easy to use and timesaving for the teachers. The poorer inter-rater reliability for REFLECT could be an indication that MaReS is easier to use. Mean rating times for MaReS were longer than for REFLECT. This probably reflects the fact that MaReS has a greater number of items (n=12) than REFLECT (n=6). In our opinion less than 15 minutes to read and rate a reflective essay (mean rating time) are still to be considered reasonable. 

### 4.2. Evidence for construct validity

In this study, we gathered validity evidence referring to the following components of construct validity: content, response process, internal structure and relationship to other variables [1], [12], [38]. MaReS might be a suitable rubric for teaching reflection and providing feedback to students written reflections. The combination of guiding questions for written reflection and the use of MaReS as a time-saving way to provide structured feedback was successfully piloted.

#### 4.2.1. Content

Validity evidence based on test content derives from an analysis of the relationship of a test content and the construct it is intended to measure [1]. The content of MaReS (including guiding questions, rubric and rater manual) was selected carefully based on a widely accepted model for the concept of the process of reflection [29]. We also considered the content of three existing tools to assess reflective essays [44], [50], [57]. More validity evidence was obtained by consulting six external experts to judge the relationship of parts of the test and the construct [1]. We also incorporated their feedback in the final version of MaReS. 

#### 4.2.2. Response process

Validity evidence based on the response process can include the response process of the raters as well as the response process of the students taking the assignment [1]. 

To support the assessment process of the raters, we developed a rater manual that comprised a short description of how to rate the essay as well as descriptors and anchor examples for all items and consecutive scales. Rater training included the two-day workshop on reflection, rating an exemplary essay and a discussion of divergent results of the rating. At the end of the discussion agreement on how to rate the essays was achieved. 

Even though “interrater reliability is enhanced by training data collectors, providing them with a guide for recording their observations” [14], and also that in this study we took care to ensure that all raters understood how essays should be rated, we found a poor inter-rater reliability for most of the items (see 4.2.3.). This might indicate that rater training might not have been sufficient. 

During the response process data about the rating time was collected. This does not add to the validity evidence; it is meant to help educators in the health professions who would like to incorporate reflective writing into their curricula to assess the feasibility of the endeavour and plan resources. 

Validity evidence based on response process of the students included student format familiarity [12]. Before writing their essays, students were handed out the guiding questions and the scoring rubric. The meaning of every item and the corresponding scores was explained. Students were given two example essays and discussed the ratings in class. 

Another source of validity evidence is understandable and accurate descriptions and interpretations of the scores for students [12]. In their evaluations all eight students stated that the assignment was “fully” or “mostly understandable” and the guiding questions were rated as “very helpful” or “helpful”. 

#### 4.2.3. Internal structure

According to Fleiss an inter-rater reliability (IRR) of 0.6 and above can be considered as excellent and an IRR above 0.4 and lower than 0.6 as fair to good. IRRs lower than .4 are considered as poor [15]. This means that IRR for MaReS in our study are only acceptable for items 1 to 4 and item 9. Looking at the wording of the items, the content that is assessed in items 1 to 4 and 9 (general comprehensibility, reference to the assignment, description of situation, description of own emotions, selection of external sources) seems to be more concrete and thus easier to rate than that of the other items. Items 5 to 8 (explanation of own emotions, describing the perspective of the counterpart, relating the perspective of the counterpart to own perspective, influence of previous experiences and reflections) and items 10 to 12 (assessment of the situation, action strategy, expectations regarding the use of the future action strategy/-ies) are integral but more difficult parts of reflection. Comprehensibility on the side of the raters seems to be more subjective in these items than for the items with an acceptable IRR. For example, on the one hand the explanation of one’s own emotions can be difficult, and students might feel the matter is too personal to share in a reflective essay. On the other hand, whether an explanation is comprehensible for a rater might depend on the rater’s personal experiences. The same applies for change of perspective. Items 10 to 12 (assessment of the situation, action plan) can be difficult for raters, because there might be an assessment of the situation by the student that is comprehensible or an action strategy that is concrete, but the rater feels that it is not sufficient to handle a similar situation better in the future. This might cause some of the raters to give lower scores. During the assessment of the essays the raters took notes on items that were difficult to rate. The analysis of these notes is not part of this study but will help to refine the rater manual and address important aspects in future rater training. 

Item 7 (relating the perspective of the counterpart to own perspective) and item 8 (influence of previous experiences and reflection) also received comparatively low ratings. This might indicate that these items are too difficult. In the case of item 7 students might need more explanation, examples and training. A reason for low scores for item 8 might be that the students in our study did not have much clinical experience and it was difficult for them to draw on previous experiences or reflections.

All IRRs that we found for the REFLECT rubric must be considered as poor. While three studies have found high IRRs for REFLECT [39], [40], [57], another study was also not able to replicate these high IRRs. The authors of the latter study state that the difference in the findings could originate from the context in which validity evidence is collected (e.g. different institution and study population) [18]. Since in the case of our study, German native speakers applied an English tool, language problems should also be considered. It is also likely that rater training for REFLECT was not sufficient. There is a short description of the application of REFLECT in the original paper [57], but the raters did not have access to a rater manual. Our rater training for REFLECT consisted of the rating and discussion of only one example essay. Miller-Kuhlman et al. applied a sounder approach for their rater training: In a training cycle the raters compared scores and discussed discrepancies for several sample essays, then rated more samples until an IRR of at least 0.8 was achieved before collecting the data for their study. This training required six hours for REFLECT [39]. 

#### 4.2.4. Relationship to other variables

Comparing a newly developed test to a test hypothesized to measure the same construct, is an important source of construct validity [1]. The authors found a positive correlation for the comparison of the MaReS scores with a global rating of the essays and with the REFLECT rubric, which is used to assess students’ reflective levels and is meant to provide individualized written feedback to guide reflective capacity promotion [57]. For the first set of essays, we found strong and significant positive correlations [8] for MaReS and REFLECT as well as for REFLECT and the global rating. For the second set we found strong and significant positive correlations for MaReS and the global rating as well as for REFLECT and the global rating. We found near moderate and moderate positive (defined by Cohen as an r of .3 [8]) correlation for MaReS/global rating in the first set of essays and MaReS/REFLECT in the second set of essays, which were both not significant probably due to the small sample size. The fact that different correlations were found in the first and second rounds could have been caused by a change of raters. Nevertheless, the results suggest that MaReS measures the same construct as the REFLECT rubric [1], namely the students’ reflective level. 

#### 4.2.5. Evaluation

This study examines data for level 1 (reaction) of the New World Kirkpatrick Model [26]. When looking at the results one must have in mind that the number of students that filled in the evaluation for MaReS was very small (n=8), and conclusions must be drawn with caution. 

Some students share very personal stories in their reflective essays, and care must be taken that feedback on the essays will not be judgmental [21] and hurt students’ feelings. The content of reflection is subjective, which is why Koole et al. suggest that assessment should focus on generic process skills [29]. Therefore, most of the rubrics that are used for assessing reflective essays (including MaReS) focus on the process of reflection rather than on the handling of a situation. Nevertheless, some students seem to feel the need for a comment on the situation, as one student mentioned that the feedback had no impact on him/her because it was only evaluating the structure of the text and not the situation itself. Teachers should also be aware that essays can contain very personal content from the students. For example, descriptions of emotional burdens, illnesses or traumatic experiences could be included in the reports. Therefore, teachers should think in advance about where support may be available and how they will react in such cases.

In effective feedback, information about previous performance is used to promote positive and desirable development [2]. Students found the feedback that they received with MaReS constructive, helpful, and non-judgmental. 

Gaining evidence on level 2 of the New World Kirkpatrick Model [26] will be challenging for MaReS, because reflective capacity is context dependent. Moniz et al. infer from their study about the use of reflective writing for student assessment that drawing meaningful conclusions about reflective capacity requires approximately 14 writing samples per student, which are each assessed by four or five raters [40]. This conclusion raises questions about the feasibility of the summative assessment of reflective writing. Results of our study also lead in the same direction: Even though the feedback instrument was mostly rated positive, half of the students felt slightly less confident when writing their second essay – and their free responses indicated that this feeling was related to the situation they chose for the second reflection (e.g., problems finding a topic for the second reflection). 

## 5. Conclusion

The Magdeburg Reflective Writing Feedback and Scoring Rubric (MaReS) can be used as a tool to guide students’ reflective writing and provide structured feedback in health professions education. In this study IRR was low for seven of the twelve items. We theorize that the rater training – consisting of the rating and discussion of one exemplary essay – was not sufficient. Using more essays for a rater training and more training cycles are likely to result in higher IRRs. A mean rating time of 13.9 minutes seems feasible and might be shorter, when raters gain more experience. If educators would like to incorporate reflective writing and its assessment into a curriculum, sufficient time for rater training must be allocated when planning resources. Caution must be taken, when summative assessment is used for reflective writing regardless of the tool that is used, because there is low predictability from one essay to the next.

The small number of students that provided feedback on MaReS considered the instrument as comprehensible and helpful. More studies with a greater number of students will be needed to support these findings. Gathering evidence for Kirkpatrick Levels of two and higher will be challenging because of the context specificity of reflective capacity. 

We recommend MaReS as a tool for teaching and formatively assessing written reflections of students in health professions education, for example on clinical experiences during a practical year, clinical rotations or block training, but also on learning experiences in general. 

## Authors’ ORCIDs

- Anja Härtl: [0009-0008-0818-6213]
- Stefan Rüttermann: [0000-0002-2293-8089]
- Linn Hempel: [0009-0009-5421-2029]

## Acknowledgements

We would like to thank the members of the Committee for Communicative and Social Competencies (KusK) and the experts for their great cooperation and support. 

## Competing interests

The authors declare that they have no competing interests. 

## Supplementary Material

Magdeburg reflective writing feedback and scoring rubric

## Figures and Tables

**Table 1:**
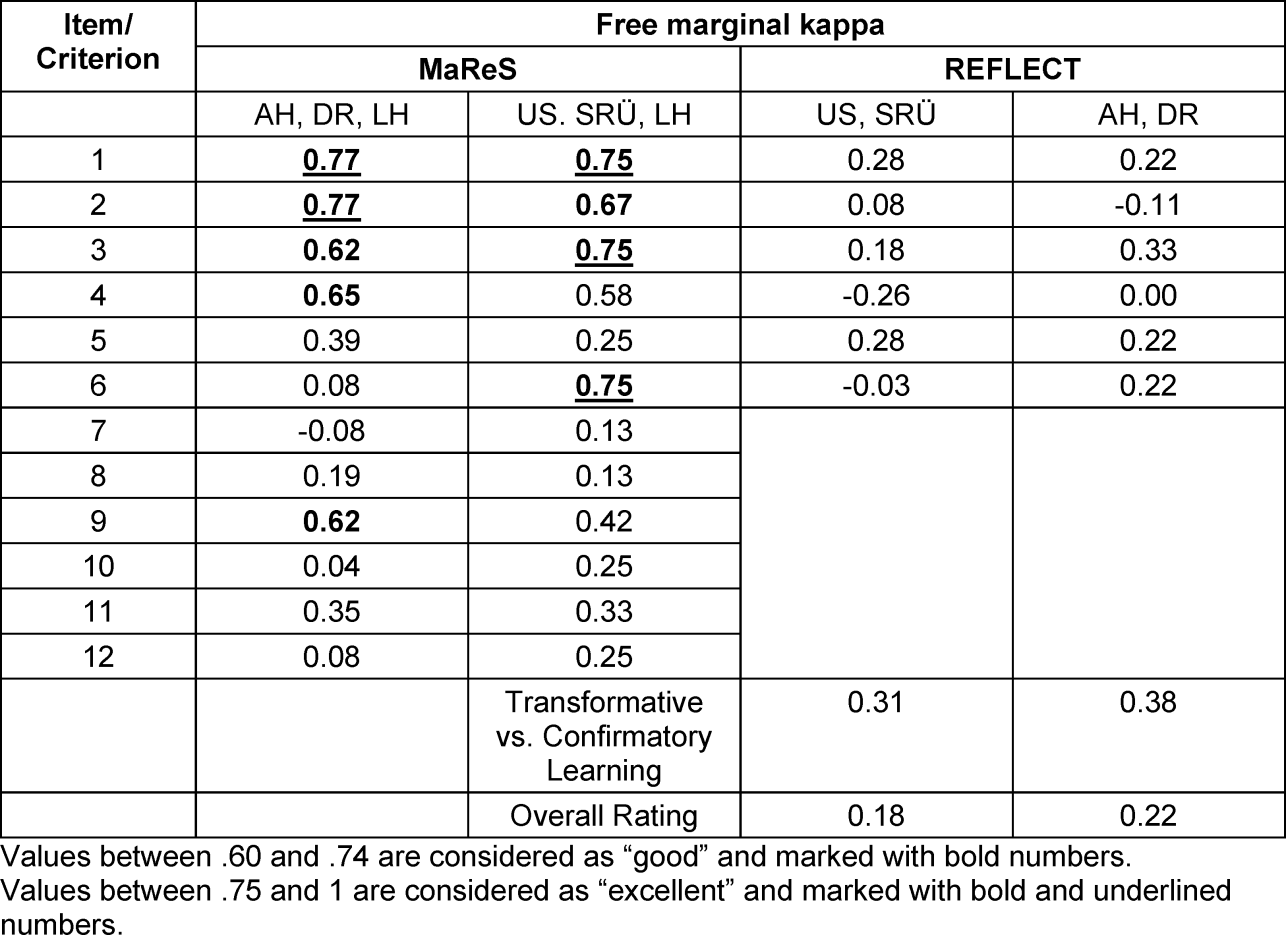
Inter-rater reliability for MaReS and REFLECT for the different raters

**Table 2:**
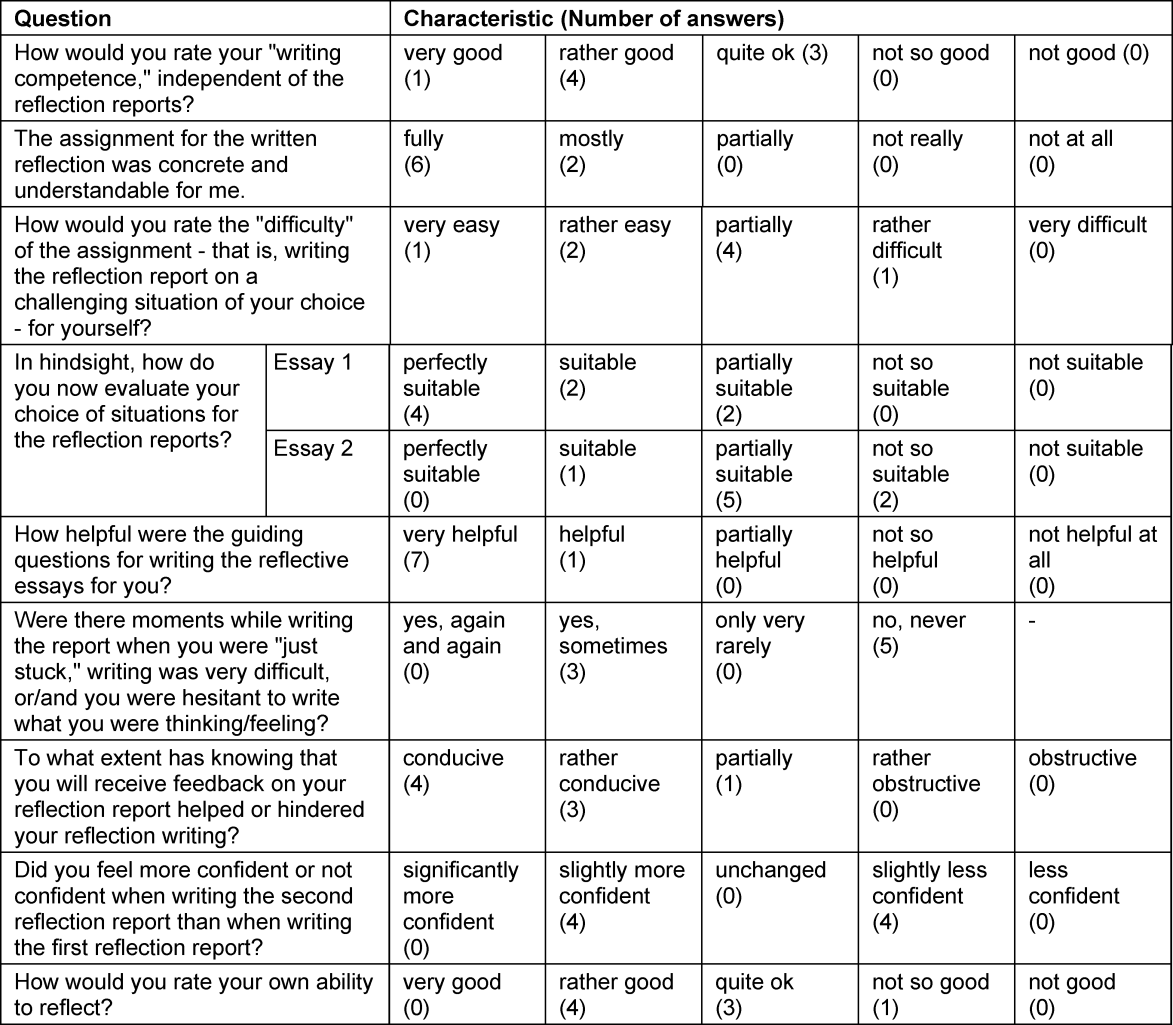
Answers to closed questions of the students’ evaluation

**Table 3:**
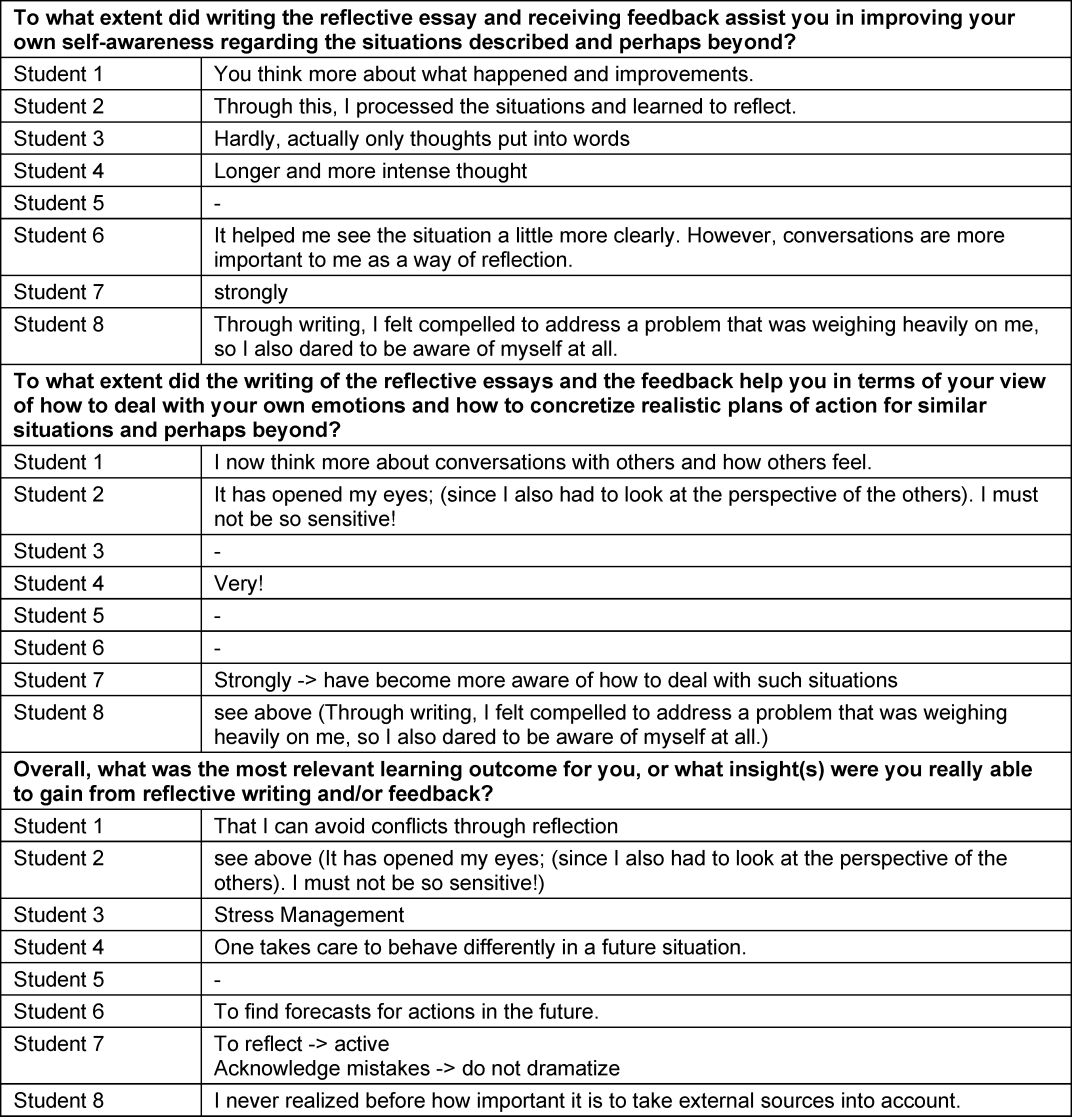
Free-text responses on the impact of writing and feedback on reflection reports
