# Supplementary material for: MaReS (Magdeburg Reflective Writing Scoring Rubric for Feedback) – development of a feedback method for reflective writing in health professions education: A pilot study in veterinary medicine
Source: GMS J Med Educ. 2025 Apr 15;42(2):Doc28. doi: 10.3205/zma001752 (PMC12131509; doi:10.3205/zma001752)
Supplement: Magdeburg reflective writing feedback and scoring rubric [file JME-42-28-s-001.pdf]

Attachment 1: Magdeburg reflective writing feedback and scoring rubric (task, guiding questions and scoring rubric)

Assignment:

Please write a reflective essay about a concrete situation. Choose a situation connected to your studies or occupation in which you have felt challenged during the interaction with a patient, a patient owner, a fellow student or a teacher.

Elaboration:

1) Scope: one to a maximum four pages (about 2,000 to a maximum of 8,000 characters without spaces)
2) Necessary information on the reflective essay:
   - Your first name and surname
   - Degree program
   - Semester
   - Number of reflective essays already submitted
   - Setting (course, internship, clinic, practice, block internship, ...)

Key questions for the reflective essay

- What specific situation or challenge do you refer to in your reflective essay? Try to be as neutral as possible.
- What emotions did you perceive in the situation and how do you explain the occurrence or the emergence of these emotions?
- How could other persons that were involved have perceived this situation? How does this view relate to your own perspective?
- What influence did your experience or previous reflections have on the course of the situation?
- What different external sources (such as peer or teacher feedback and literature review) could you use to assess the situation and draw conclusions for the future?
- How do you assess the overall experience based on the previous reflection?
- Which action strategy (s) would be suitable for you for similar situations in the future? What consequences could result from using your future action strategy (s)?

Attachment 1 to Ramspott S, Sonntag U, Härtl A, Rüttermann S, Roller D, Giesler M, Hempel L. *MaReS (Magdeburg Reflective Writing Scoring Rubric for Feedback) – development of a feedback method for reflective writing in health professions education: A pilot study in veterinary medicine.* GMS J Med Educ. 2025;42(2):Doc28. DOI: 10.3205/zma001752

Feedback on the reflective essay

First name, surname:
Degree program:
Semester:
Number of reflective essays already submitted:
Setting (course, internship, clinic, practice, block internship, ...):

Item	Assessment based on the coding guide: Points each ☐ 2 ☐ 1 ☐ 0, please mark clearly: ☒. If you want to correct the selected, please underline it clearly. If you want to return to the previous selection after a correction, please underline the selection that should apply.	Score:
1	**General comprehensibility:** ☐fully comprehensible ☐partially comprehensible ☐difficult to comprehend	
2	**Reference to the task:** ☐clearly recognizable ☐partially recognizable ☐hardly recognizable/not present	
3	**Description of the situation:** ☐fully comprehensible ☐partially comprehensible ☐difficult to comprehend/not present	
4	**Description of own emotions:** ☐fully comprehensible ☐partially comprehensible ☐difficult to comprehend/not present	
5	**Explanation of own emotions:** ☐fully comprehensible ☐partially comprehensible ☐difficult to comprehend/not present	
6	**Describing the perspective of the counterpart:** ☐fully comprehensible ☐partially comprehensible ☐difficult to comprehend/not present	
7	**Relating the perspective of the counterpart to own perspective:** ☐fully comprehensible ☐partially comprehensible ☐difficult to comprehend/not present	
8	**Influence of previous experiences and reflections:** ☐fully comprehensible ☐partially comprehensible ☐difficult to comprehend/not present	
9	**Selection of external sources (e.g. feedback, literature):** ☐more than one source, sources of different origin ☐only one source or sources of same origin ☐ no sources included	
10	**Assessment of the situation:** ☐fully comprehensible ☐partially comprehensible ☐difficult to comprehend/not present	
11	**Action strategy:** ☐concrete ☐not concrete ☐not present	
12	**Expectations regarding the use of the future action strategy(s):** ☐fully comprehensible ☐partially comprehensible ☐difficult to comprehend/not present	
	**Total score (max. 24)**	

Attachment 1 to Ramspott S, Sonntag U, Härtl A, Rüttermann S, Roller D, Giesler M, Hempel L. *MaReS (Magdeburg Reflective Writing Scoring Rubric for Feedback) – development of a feedback method for reflective writing in health professions education: A pilot study in veterinary medicine.* GMS J Med Educ. 2025;42(2):Doc28. DOI: 10.3205/zma001752
